# Dental Irritation as a Factor in the Causation of Epilepsy

**Published:** 1888-03

**Authors:** 


### SELECTIONS.

### Dental Irritation as a Factor in the Causation of Epilepsy.

Read before the Philadelphia Neuralogical Society, December 19th, 1887.

The author of the following paper, after some general remarks on the nature, peculiarities and causes of epilepsy; presents a number of exceedingly interesting and instructive cases, showing the influence of diseased teeth, in the production of epilepsy; and the prompt and permanent relief afforded by the removal of the offending tooth or teeth.

The influence of diseased teeth in the production of various phases of disease, not only of a local but of a more general character, has been too much overlooked—has not received the attention its importance merits. It is a happy sign that it is receiving more attention. ED. REG.

The object of this paper is to direct the attention of physicians to a cause of epilepsy which has not hitherto been estimated at its full value, inasmuch as in none of the standard works upon neurology is the subject even alluded to, viz: pathological state of the dental structures. That dental inflammations and disorders are more often provocative of epileptic seizures than is commonly supposed appears quite certain from the following cases, and also from the character of the cause and its effect. Many reasons might be given why dental disorders are peculiarly adapted to call forth this periodical discharge, and why these disorders are habitually overlooked by the physician, but they need not be detailed here. As exemplifying these phenomena, some interesting and instructive cases are adduced.

The following case occurred in the clinical service of Dr. Wharton Sinkler, at the Orthopædic Hospital and Infirmary for Nervous Diseases, to whose kindness I am indebted for the privilege of recording it:

*Case I.*—Mary L., aged 9, was brought to the hospital in October, 1886, with a history of epilepsy dating from May of the same year. The convulsive attacks first made their appearance on the afternoon of the same day that the child had had three teeth extracted on account of repeated attacks of toothache. One decayed tooth, however, was left remaining in the lower jaw.

Previous to coming to the hospital, the epileptic attacks occured two and three times a week. From all that could be learned from the mother, the symptoms were those of a typical epilepsy. There was no neurotic history in the family. She was placed upon from three to five drops of the fluid extract of cannabis indica for two weeks, during which period she had twelve attacks. The bromide of sodium was then given for two weeks, during which she had sixteen attacks. From November 1 to March 1, 1887, she continued taking the bromides alone, in combination, and, finally, in conjunction with the infusion of digitalis. During the four months of steady treatment she had forty seizures. About the first of March, the mother made the remark that the child was always extremely restless at night; that she would lie awake for hours complaining of toothache; and even when asleep she would continually grind her lower teeth against the upper teeth. Examination of the mouth revealed a carious and inflamed condition of a molar tooth in the lower jaw on the left side.

From the history of the case, and the possibility that the irritation arising from the diseased tooth might be the exciting cause of the attack, it was concluded to have the tooth removed. This was done under the influence of nitrous oxide gas. The night following the child rested much better, and from that time forward her sleep became natural, her appetite improved, and her general health became decidedly better. From the last week in February until the present time (December 19, 1887) she has not had a single symptom of an epileptic attack.

That a dental irritation should be capable of exciting an epileptic condition does not appear at all strange, when it is fully comprehended how numerous are the recorded cases of ocular, aural, visceral, muscular, and nervous disorders which have been caused by the irritation arising from the pathological conditions of the teeth and associated structures.

The interest aroused by the result of the preceding case led to an examination of medical literature for reports of similar cases. I find that no less than sixteen cases, entirely and immediately cured by the removal of an irritating tooth, have been recorded by different observers, and which are here arranged in chronological order. It is not supposed that this collection embraces all the recorded cases, but it is hoped that it will elicit references to many others, and, what is more important, the reporting of many new cases.

The injurious effects of diseased teeth, and the irritation arising from them, in the production of many general diseases, did not escape the acute mind of Dr. Rush. In a paper published in his collected works[1] he records the following:

*Case II.*—"Some time in the year 1801, I was consulted by the father of a young gentleman in Baltimore who had been afflicted with epilepsy. I inquired into the state of his teeth, and was informed that several of them in his upper jaw were much decayed. I directed them to be extracted, and advised him afterwards to lose a few ounces of blood at any time when he felt the premonitory symptoms of a recurrence of the fits. He followed my advice, in consequence of which I had lately the pleasure of hearing from his brother that he was perfectly cured."

Dr. Ashburner published,[2] in 1834, a number of remarkable cases of hysteria, spasms, convulsions, etc., due to diseased conditions of the teeth. Among others was the following case of epilepsy:

*Case III.*—A young lady of highly nervous temperament was attacked with epilepsy in the eighth month of her first pregnancy. She had two attacks before her labor, which was a very favorable one. Seven months afterwards the fits reappeared, and occurred two and three times a week. Various methods of treatment were resorted to without success. For a while the intervals between the attacks were somewhat longer, and for a while they appeared twice daily. An examination of the mouth revealed seven carious teeth, which were at once removed. Three wisdom teeth were prevented from erupting on account of a cartilaginous condition of the gums. These obstacles were removed.

1 Enquiries and Observations, vol. i, p. 199.
2 On Dentition and some Coincident Disorders, p. 98.

The epileptic fits at once ceased, and after several years they had not returned.

*Case IV.*—Albrecht relates[3] the case of a boy, aged 12 years, who for a period of six months suffered daily with general convulsive attacks. Just preceding the attack there was severe pain in the temporal region. No cause could be assigned for the seizures. Treatment was without avail. Examination of the mouth revealed an overcrowded condition of the teeth, which were in addition unusually large. After removal of some of the teeth the convulsions subsided, and in a short time entirely disappeared.

*Case V.*—Dr. Tomes publishes[4] the following: "A lad, a farm-laborer from Windsor, was admitted into the hospital for epilepsy. The usual remedies were tried for six weeks without effect. His mouth was then examined, and the molar teeth of the lower jaw were found to be much decayed, and of some of these only the fangs remained. He did not complain of pain in the diseased teeth, or in the jaw. The decayed teeth were, however, removed, and the fangs of each were found to be enlarged and bulbous from exostosis. During the eighteen months that succeeded the removal of the diseased teeth, he had not suffered from a single fit, though for many weeks previous to the operation he had two or three per day."

*Case VI.*—Dr. Baly records[5] the history of a case of epilepsy from dental irritation, occurring in a man, aged 45. The patient was an employe in the Millbank Penitentiary; was of good physique; in good health, and had never suffered from vertigo, headache, or any form of nervous trouble. In the latter part of October, 1850, he began to suffer from toothache. On November 4, the tooth was examined by the medical officer, but on account of its carious condition and deficient light it was not extracted. Nitric acid, however, was applied, which gave the patient relief. On the 6th the muscles of the right side of the face began to twitch. The muscular spasms lasted four or five minutes, and occurred three or four times a day.

"At these times, when the twitchings had reached a certain

3 Casper's *Wochenschrift*. 1837, p. 125.

4 System of Dental Surgery.

5 *London Med. Gazette*, xlviii, p. 534-540.

degree of intensity, the jaw became locked, and he lost the power of speech; but he had no pain in the head, giddiness, or sense of stupor. The paroxysm of spasm in the muscles of the right side of the face and jaws recurred the next day, and on the following day, the fourth after the examination by Mr. Chatfield (the medical officer), the twitchings became more violent, and his jaw locked. He had the sensation of all his teeth falling out, and then lost consciousness. A strong convulsive fit ensued, which lasted half an hour; the same night he had a second fit." These attacks were described as presenting all the features of an epilepsy. A third attack occurred before morning. The next day the tooth was extracted, together with a small piece of bone attached to the root.

For one month the patient was perfectly well, but on the 7th of December, in the middle of the day, he again experienced the spasmodic twitchings, and at the same time became conscious of the existence of something protruding from his jaw; with his fingers he removed a piece of dead bone. In the evening of the the same day the spasmodic contractions of the face occurred several times. On the night of December 8, he awoke with a spasm in the cheek, and upon getting out of bed fell upon the floor unconscious; a general convulsive fit followed, during which there was foaming from the nose and mouth. At 6 A. M. a second fit followed, more violent than the first, and lasted five minutes. In the intervals of these attacks there was considerable uneasiness and confusion of mind. The next night he suffered a return of the fit. Examination of the mouth revealed a swollen and tumid condition of the gum, but there was no discernable source of irritation. The patient was placed on calomel to prevent further mischief to the deeper-lying structures around the diseased tooth-socket. He remained well until February 22, when he had, for the space of ten minutes, the same premonitory twitchings in the muscles of the face, but no real fit. A small piece of dead bone was extracted from the gum, after which the old wound healed, and the patient entirely recovered.

*Case VII.*—Dr. Ramskill publishes[2] the following: "A boy,

2 *Med. Times and Gazette*, 1862, vol. ii, p. 216.

13 years old, has had frequent attacks of epilepsy for the last eighteen months. Latterly, his mother noticed that some days he rubs his left cheek, complaining of face-ache, after which the fit follows. On examining the mouth there is to be seen a molar tooth considerably decayed, with a swollen gum around it and partly growing over into the cavity; it is not very tender to the touch, and the examination does not give rise to toothache. On questioning, I find the sensation which the boy experiences before the fit does not seem to be one of pain, but rather indefinite uneasiness. He always has a fit the night this comes on. He never felt it during the day; it is always about seven or eight o'clock. I desired the mother to have the tooth extracted, and ordered a simple saline, with $\frac{1}{4}$ grain of belladonna, to be taken twice daily. This was in June. The tooth was extracted next day. I saw this boy once a fortnight from that time for four months, but he had no recurrence of the fits. In this case I believe an unfelt aura commenced about the gum surrounding the tooth, and was not recognized till some degree of inflammation arose, and thus a modification of pain became associated with the aura, and directed attention to it."

*Case VIII.*—Trosseau relates[3] the case of a patient, a young notary's clerk, under the care of Dr. Foville, who had been subject to monthly attacks of epilepsy for several years. Many remedies had been tried in vain. Dr. Foville suggested the extraction of some carious teeth which ached constantly. The suggestion was acted upon, and from that day the fits disappeared.

*Case IX.*—W. H. Waite reports[5] the case of a young woman, aged 18, who consulted him for treatment of a carious condition of the incisor and canine teeth of the upper and lower jaws. The teeth had been diseased for four years, and were very sensitive. For three years the patient had been subject to epileptic attacks, which were at first quite slight, but had gradually increased in severity. After removal of the diseased teeth, and filling of others, the epileptic fits entirely ceased. After some months the fits returned, attended with sharp, shooting pains in the alveolus. Examination showed that several other teeth had

3 *Clinical Medicine*, New Sydenham Soc., vol. i.
5 *British Journal of Dental Science*, 1863.

become decayed. These were removed, and from that time on there was no recurrence of the epilepsy, and the patient increased in health and weight.

*Case X.*—Dr. Nathan Field reports[6] the case of a boy, about 5 years old, who was suddenly seized with an epileptic fit. In two weeks he had a second attack, which passed away after a few minutes. In the course of the next ten days it was estimated that the boy had a thousand convulsions, occurring every few minutes. No cause could be assigned. It was finally observed that before the appearance of the convulsion there was a twitching of the muscles of the left side of the face. Finally, after a severe convulsion, while the child was unconscious, he drew up his upper lip, when it was observed that the canine tooth had, instead of causing absorption of the deciduous tooth, pushed it outward through the alveolus, the gum, and into the lip. The tooth was removed, and in less than an hour the convulsions subsided and never appeared again.

*Case XI.*—Mr. Canton related[7] the history of the following case: A strong, healthy boy, aged 19, who had become the subject of epileptic fits, applied to Mr. Canton for treatment. As the cause of the fits could not be ascertained, it occurred to him that they might be due to the eruption of a wisdom tooth. The gum was freely incised, and the crown of the tooth laid bare. From that time the fits never returned.

*Case XII.*—Mr. Henry Moon related[1] the following case: "The patient, a girl, aged 21, was brought as an out-patient to Dr. Fagge, at Guy's Hospital, and he, finding that her teeth were in a very bad state, sent her to Mr. Moon. She had suffered from fits since she was fourteen, and lately they had become so frequent as to reduce her almost to the condition of imbecility. On examining her mouth, a third molar was found in process of eruption; this he lanced freely. Some carious teeth were extracted and others were filled. Treatment by the bromides of potassium was ordered at the same time. The result was that the fits entirely ceased from the day of her first visit to

6 *Western Journal of Medicine*, 1869.
7 *Proceedings Odontological Soc. of Great Britain*, 1880.
1 *Proceedings Odontological Soc. of Great Britain*, 1882.

the hospital. The girl recovered her intellect, and although she was kept under observation for several months, she had no return of the fits."

*Case XIII.*—Dr. Schwartzkopf reported[2] the following case in the *Deutsche Monatschrift fur Zahnheilkunde*, 1886: "A man, aged 27, suffered severe pain in the right upper central incisor, which was carious, and consulted a dentist, who filled it. Soon after this a swelling appeared in the hard palate, where an opening formed. The patient was now easy, but the tooth continued loose and tender when touched. The fistula also remained patent and discharging. Ten days after the tooth was filled the patient had an epileptic attack, and these recurred at gradually shorter intervals, until, at the end of eighteen months, they occurred several times a week. During this time the patient was treated with bromides, atropine, etc., but without results. The tooth was then extracted, the fistula healed, and the fits ceased, and at the time of reporting, the patient had remained free from them for four years."

The two following cases are reported[3] by Dr. Liebert:

*Case XIV.*—Emil S., aged 25, in good health and with no neurotic tendency, began to suffer with attacks of vertigo in February, 1883. These attacks lasted several minutes, after which the patient appeared perfectly well. On one occasion, however, the vertigo was so severe that he was compelled to sit down to keep from falling. On one occasion he lost consciousness. By April 25 the attacks had greatly increased in severity. On this day he had such a severe epileptic attack that Dr. Liebert was called in. The patient had been lying on the floor for fifteen minutes wholly unconcious, and most of the muscles of the body in a state of tonic contraction; the pupils were of medium width and insensible to light; there was also a fresh wound of the tongue. After careful inquiry, it was learned that just previous to the attacks the patient experienced a peculiar tickling or crawling sensation in the tongue, an inability to speak words distinctly, and some involuntary movements of the tongue. Immediately after there followed the giddiness, the fall, unconscious-

2 *Journal British Dental Assoc.*, 1886

3 *Deutsche medicin. Wochengchrift*, September, 1885.

ness, etc. Despite large doses of the bromides, the attacks increased in frequency and severity. Finally, in June, he began to suffer with toothache. Examination of the mouth revealed several carious teeth, one of which was very sensitive to percussion. This was extracted, and from that moment all peculiar sensations and motions of the tongue ceased, and there has not been in the past two years a single epileptic seizure. This patient had in the four months several hundred attacks of vertigo and eighteen or twenty typical epileptic convulsions.

*Case XV.*—Young man, aged 35, cabinet maker. Began having epileptic attacks on February 3, 1862, which came on almost daily and with increasing severity. On March 5 he had twenty-three seizures. With the exception of toothache he had never been sick. Repeated inquiries elicited the information that from December, 1861, the use of his tongue was for some seconds, or even minutes, frequently rendered difficult, and this fact was coupled with a certain feeling of illness or virtigo. In the attack of February 3, 1862, these symptoms were exceptionally severe, the tongue being drawn to the right side and executing spasmodic movements. Immediately thereafter he became unconscious and fell to the floor in convulsions. The tongue symptoms were usually premonitory of the frequent subsequent attacks. Owing to the fact that the aura appeared to be connected with the mouth, it was determined to seek for the cause in that locality. As he had had toothache occasionally, several carious teeth were removed. The patient at once declared that he felt an unwonted freedom from a former oppressive feeling, and that he believed he would have no more of the seizures. His conjecture was correct, for he remained free from them from that time forth. This patient had epileptoid virtigo for three or four months, and severe epileptic attacks for thirty-eight days.—*Med. and Surg. Reporter.*

A simple method of preserving fresh fruit is to place it in a wide-mouth, glass stoppered bottle with a little chloroform. The stopper of the bottle should be greased with a little petrolatum to make it air-tight. A drachm of chloroform suffices for a quart bottle. The chloroform soon evaporates on exposure to the air, or is dissipated in cooking.—*Phar. Era.*

